# Antihypertensive and Antifibrosis Effects of Acupuncture at PC6 Acupoints in Spontaneously Hypertensive Rats and the Underlying Mechanisms

**DOI:** 10.3389/fphys.2020.00734

**Published:** 2020-08-26

**Authors:** Juan-Juan Xin, Qiu-Fu Dai, Feng-Yan Lu, Yu-Xue Zhao, Qun Liu, Jing-Jing Cui, Dong-Sheng Xu, Wan-Zhu Bai, Xiang-Hong Jing, Jun-Hong Gao, Xiao-Chun Yu

**Affiliations:** Institute of Acupuncture and Moxibustion, China Academy of Chinese Medical Sciences, Beijing, China

**Keywords:** electroacupuncture, spontaneously hypertensive rats, myocardial fibrosis, angiotensin II, TGF-β_1_, TNF-α

## Abstract

Long-term hypertension can lead to both structural and functional impairments of the myocardium. Reversing left ventricular (LV) myocardial fibrosis has been considered as a key goal for curing chronic hypertension and has been a hot field of research in recent years. The aim of the present work is to investigate the effect of electroacupuncture (EA) at PC6 on hypertension-induced myocardial fibrosis in spontaneously hypertensive rats (SHRs). Thirty SHRs were randomized into model, SHR + EA, and SHR + Sham EA groups with WKY rats as a normal control. EA was applied once a day for 8 consecutive weeks. The cardiac fibrosis as well as the underlying mechanisms were investigated. After 8 weeks of EA treatment at PC6, the enhanced myocardial fibrosis in SHRs was characterized by an increased ratio of left ventricular mass index (LVMI), collagen volume fraction (CVF), and elevated content of hydroxyproline (Hyp) as well as the upregulated expression of collagen I and collagen III in myocardium tissue of SHRs. All these abnormal alterations in the SHR + EA group were significantly lower compared to the model group. In addition, EA at PC6 significantly improved the pathological changes of myocardial morphology. Meanwhile, the increased levels of angiotensin II (Ang II) and tumor necrosis factor-α (TNFα) and expression of transforming growth factor β1 (TGF-β1), connective tissue growth factor (CTGF), matrix metalloproteinase (MMP)-2, and MMP-9 in the serum or heart tissue of SHRs were also markedly diminished by EA. These results suggest that EA at bilateral PC6 could ameliorate cardiac fibrosis in SHRs, which might be mediated by the regulation of the Ang II – TGF-β_1_ pathway.

## Introduction

Hypertension is a prevalent cardiovascular disorder that affects more than one billion people worldwide. Prolonged elevated blood pressure often leads to cardiac hypertrophic and myocardial fibrotic alterations, which are determinant for the development of heart failure, myocardial infarction, and stroke (Lewington et al., 2016). Therefore, the pathological progress from hypertension to cardiac hypertrophy and myocardial fibrosis becomes the pivotal issue to address and turns to be a great challenge in clinical management (Diez, 2014). Herein, myocardial fibrosis is known to occur in patients with left ventricular hypertrophy (LVH) caused by persistently elevated blood pressure (Varo et al., 2000) and contributes critically to an increased risk of adverse cardiac events (Zile et al., 2015). It is mainly characterized by excessive accumulation of extracellular matrix (ECM) in the myocardium (Berk et al., 2007) and is deemed as an indicator of mortality in patients with failing myocardium (Lopez et al., 2015). Therefore, reversing or alleviating cardiac fibrosis by adequate blood pressure control would be a major therapeutic goal for patients to avoid the occurrence of heart failure.

Increasing evidence has suggested that both exaggerated collagen (type I and type III) synthesis and inadequate collagen degradation result in the accumulation of ventricular collagen (Varo et al., 2000) in the remodeling fibrotic interstitium of heart (Azevedo et al., 2016). In addition, the rate-limiting step in the extracellular degradation of collagen is the catalytic cleavage associated with the balance between MMPs and their inhibitors (Berk et al., 2007). The myocardial fibrosis can be myocyte death, inflammation, enhanced workload, or hypertrophy and is often aggravated by the renin-angiotensin system (RAS), cytokines (TNF-α), growth factors (such as TGF-β1 and CTGF), and matricellular proteins. Extensive evidence implicates that neuro-hormonal pathways play crucial roles in the pathogenesis of cardiac fibrosis, in which the function of angiotensin II (Ang II) has been highly emphasized due to its direct actions (Wu et al., 2002) and indirect contribution through TGF-β_1_ (Chung et al., 2014). TGF-β_1_ is ubiquitously expressed in the cardiovascular system (Biernacka et al., 2011), which can trigger the differentiation of cardiac fibroblasts to activated myofibroblasts and may directly participate in promoting cardiac fibrosis in experimental models (Dobaczewski et al., 2011) as well as in fibrotic human hearts (Mancini et al., 2018). In addition, TNF-α has been shown to regulate TGFβ isoforms by decreasing collagen synthesis in cardiac fibroblasts (Sivasubramanian et al., 2001) and increase MMP expression by reducing synthesis of inhibitors of metalloproteinases (Sun et al., 2007). To date, considerable progress has been made in antifibrotic activities of established drugs and novel compounds, including Ang-II pathway inhibitors (De Mello and Specht, 2006), TGF-β pathway inhibitors (Tank et al., 2014), and matrix metalloproteinase inhibitors (Spinale, 2007). Although these interventions may lead to a partial recovery of contractile function via a process known as “reverse remodeling” and thereby delay the progression toward heart failure (Gourdie et al., 2016), the individual and societal costs of heart diseases linked to fibrosis are staggering (Kohl and Gourdie, 2014). Notably, previous clinical studies have revealed that acupuncture is a stand-alone or adjunctive therapy in blood pressure control and treatment of various cardiovascular illnesses (Li et al., 2015). It has been demonstrated that Ang II (Shi et al., 2017), cytokines (TNF-α) (Murakami et al., 2019), growth factors (such as TGF-β_1_ and CTGF) (Murakami et al., 2019), and matricellular proteins (Lin et al., 2016) could be modulated by EA treatment. However, the definitive mechanisms underlying antihypertensive and antifibrosis responses of EA are still unclear.

Increasing experimental and clinical studies show that EA at the PC6 acupoint produces therapeutic effects on the treatment of cardiovascular diseases (Li et al., 2015; Shi et al., 2017). Our previous work demonstrates the beneficial role of ACE, AT1R, and AT2R against hypertension-induced cardiac hypertrophy by EA applied at the bilateral PC6 acupoints (Xin et al., 2017). In the present study, using spontaneously hypertensive rats (SHRs), we sought to investigate EA’s effect on the synthesis and deposition of collagen stimulated by profibrotic factors so as to explore the antifibrosis effects of acupuncture and its underlying mechanisms. Specifically, the possible roles of the AngII-TGFβ_1_ pathway in the mediation of the inhibitory effects of EA (8 weeks) on hypertension and myocardial fibrosis were investigated in our present study.

## Materials and Methods

### Animal Preparation

Thirty-six male SHRs at the age of 12 weeks and 12 male Wistar-Kyoto (WKY) rats of the same age, weighing 240–270 g, were obtained from Vital River Laboratories (Certificate NO. SCXK 2012–0001, Beijing, China). The rats were housed in cages at 24 ± 1°C and humidity of 50 ± 5% under a 12-h light/dark cycle and received standard diet and water *ad libitum*. The study was carried out adhering to guidelines provided by National Institutes of Health for the Care and Use of Laboratory Animals and all efforts were made to minimize suffering of animals.

### Animal Grouping and Electroacupuncture Treatment

The rats were randomly divided into four groups: WKY group (*n* = 12), SHR group (*n* = 12), SHR + EA group (*n* = 12), and SHR + Sham group (*n* = 12). Under isoflurane inhalation anesthesia, the animals in the SHR + EA group were subjected to electroacupuncture treatment at bilateral Neiguan acupoints (PC6, located on the lateral of the lower 1/6 of the forearm between the adius and ulna). Sterilized disposable stainless steel needles (0.3 × 15 mm, Global brand, Suzhou, China) were penetrated 2 mm into the subcutis beneath the acupoints and connected with a Han’s Acupoint and Nerve Stimulator (Model HANS-200A, Ji Sheng Medical Technology Co., Ltd., Nanjing, China). Electrical stimulation (2/15 Hz, 1 mA) proceeded for 30 min per day for a period of 8 weeks (Li et al., 2010). In the SHR + Sham group, needles were inserted in the superficial layer of PC6 with no electrical stimulation applied.

### Blood Pressure Measurement

Under a conscious condition, the systolic blood pressure (SBP) was recorded by using a CODA Mouse & Rat Tail-Cuff Blood Pressure System (Kent Scientific Co., Torrington, CT, United States). The measurement was conducted once every week at 9–11 am in a quiet room. Before starting measurements, the CODA cover was lifted and the animal’s tail temperature measured by pointing the infrared thermometer at the tail’s base. Following a 10 min warm-up period, 10 preliminary cycles were performed to allow the rats to adjust to the inflating cuff. The blood pressure of each rat was tested three consecutive times to calculate the mean value.

### Assessment of Left Ventricular Mass Index

Six rats in each group were decapitated after 8 weeks of EA treatment; rat hearts were removed and washed with 4°C normal saline. After drying with filter paper, left ventricles were dissected and weighed to calculate left ventricular mass index (LVMI), which was defined as the ratio of left ventricular (LV) weight to body weight (mg/g).

### Van Gieson Staining

Three rats in each group were anesthetized by 10% urethane and transcardially perfused with 250 mL of 0.9% saline immediately followed by 300 mL of 4% paraformaldehyde in 0.1 M phosphate-buffered solution (PB, pH 7.4). The LV section was cut off transversely at the mid-ventricular level for paraffin sectioning (Chen et al., 2012). The myocardial sections (5 μm) were deparaffinized and rehydrated and stained with Van Gieson according to Brilla’s (Brilla et al., 1991) methods. In the Van Gieson–stained sections, myocardial cells were stained yellow while collagen was stained light red. The images were captured by a digital camera connected to a microscope (Pannoramic MIDI/250, 3D HISTECH, Hungary). Five randomly selected microscopic fields of each section were analyzed for collagen deposition using the Pannoramic viewer (Quant center, 3D HISTECH, Hungary), which was expressed as collagen volume fraction (CVF), the percentage of the area stained light red for collagen to the total area of each microscopic field. The CVF of each rat represents the mean of five randomly selected microscopic fields, which were measured to represent the development of myocardial fibrosis.

### Hydroxyproline Content Assay

Hydroxyproline was assayed using the chloramine T assay (Jia et al., 2010). The apex of the left ventricle was defatted and lysed. Then, the sample was centrifuged at 3000 rpm for 10 min. After centrifugation, the supernatant was mixed with fresh chloramine T for 10 min, followed by mixture with Ehrlich’s reagent at 75°C for 20 min. After samples were cooled, optical density was read at 550 nm with a spectrophotometer that was adjusted by a blank. The blank was prepared by the same procedure but without cardiac tissues in the reaction mixture. Hydroxyproline concentration, expressed as micrograms per milligram of dry heart weight, was then calculated as described before (Zhai et al., 2008).

### Tissue Preparation and Immunohistochemical Staining

After 8 weeks of EA treatment, three rats in each group were anesthetized by 10% urethane and transcardially perfused with 250 mL of 0.9% saline immediately followed by 300 mL of 4% paraformaldehyde in 0.1 M phosphate-buffered solution (PB, pH 7.4). After perfusion, the LV tissue at the mid-ventricular level was dissected out from the heart and stored in 25% sucrose PB at 4°C. A series of LV sections from rats in each group were cut at a thickness of 20 μm on a cryostat (Thermo, Microm International FSE, Germany) and divided into three groups for the three sections. The sequentially mounted slides were prepared for their respective types of fluorescent immunohistochemical and histochemical staining. Primary antibodies, including mouse monoclonal anti-TGF-β1 antibody (1:250, Abcam, Hong Kong), rabbit polyclonal anti-collagen III antibody (1:100, Abcam), rabbit polyclonal anti-CTGF antibody (1:100, Abcam), and mouse monoclonal anti-collagen I antibody (1:100, Abcam) were used in this study. Goat antimouse Alexa Fluor 488 or 594 secondary antibody (1:500, Molecular Probes, Eugene, OR, United States) and goat antirabbit Alexa Fluor 488 or 594 secondary antibody (1:500; Molecular Probes) were used to visualize the corresponding primary antibodies. Additionally, Alexa Fluor 488 phalloidin (1:1000, Molecular Probes) and 4′, 6-diamidino-2-phenylindole (DAPI, 1:40,000; Molecular Probes) were applied for counterstaining. The three groups of tissue sections were treated with fluorescent immunohistochemical and histochemical stains to examine the relationship of (1) Collagen I and Collagen III, (2) TGF-β_1_ and myocardial structure, and (3) CTGF and myocardial structure. The staining methods are as follows. After a brief washing in 0.1 M PB (pH 7.4), tissue sections were incubated in a 0.1 M PB (pH 7.4) containing 3% normal goat serum and 0.5% Triton X-100 for 30 min for blocking non-specific binding. To examine the correlation between Collagen I and Collagen III, the sections were transferred to mouse monoclonal anti-collagen I antibody (1:100, Abcam) and rabbit polyclonal anti-collagen III antibody (1:100, Abcam) to incubate overnight at 4°C. We used a similar procedure in the immunohistochemical staining to examine the correlation between TGF-β_1_ and myocardial structure as well as CTGF and myocardial structure. After incubating with blocking solution, primary mouse monoclonal anti-TGF-β1 antibody (1:250, Abcam, Hong Kong) and rabbit polyclonal anti-CTGF antibody (1:100, Abcam) were simultaneously added onto the sections for incubation overnight at 4°C. On the following day, after washing five times with 0.1 M PB, sections were exposed to goat antimouse Alexa Fluor 594 secondary antibody (1:500; Molecular Probes, Eugene, OR, United States), followed by Alexa Fluor 488 phalloidin (1:1000; Molecular Probes). After 2 h of incubation, sections were washed five times with 0.1 M PB and stained with DAPI for 5 min. After washing, the sections were coverslipped with 50% glycerin. All immunohistochemistry for each staining combination was performed at the same time to ensure the consistency of staining. During the staining process, the sections were kept inside a black container at room temperature. Samples were recorded with a confocal imaging system (FV1200, Olympus, Japan) and analyzed using the Olympus Image Processing Software. Final images were processed with Adobe photoshop CS5 and Adobe illustrator CS5 (Adobe Systems, San Jose, CA, United States).

### Enzyme-Linked Immunosorbent Assay of Ang II in Heart Tissue and TNF-αin the Serum

Six rats in each group were decapitated after 8 weeks of EA treatment; meanwhile, the blood and LV tissues were collected and centrifuged for reserve. Concentrations of Ang II in heart tissue and TNF-α in the serum were measured by ELISA following the manufacturer’s protocol (R&D, Minneapolis, MN, United States).

### Western Blotting

Heart tissues were lysed in RIPA buffer containing phosphatase and protease inhibitors (Roche Complete, Roche Diagnostics, Mannheim, Germany). The protein concentration in the supernatant was determined using the BCA method with a bovine serum albumin standard. An equal amount of total protein was subjected to SDS-PAGE and blotted on a NC membrane (Millipore, Billerica, MA, United States). The blots were blocked with 5% defatted milk powder in Tris-buffered saline (TBS) buffer and then incubated with the respective primary antibodies (mouse anti-collagen I 1:500, Abcam, United Kingdom; rabbit anti-collagen III 1:500, Abcam, United Kingdom; mouse anti-TGF-β_1_ 1:1000, Abcam, United Kingdom; rabbit anti-CTGF 1:1000, Abcam, United Kingdom; mouse anti-MMP2 1:2000, mouse anti-MMP9 1:500, Abcam, United Kingdom; mouse anti-glyceraldehyde-3-phosphate dehydrogenase (GAPDH) 1:20,000, TDY Biotech Co., Ltd., Beijing, China) overnight at 4°C. The membrane was washed with TBS and incubated with horseradish peroxidase-conjugated goat antimouse or rabbit IgG (1:10,000; TDY Biotech Co., Ltd., Beijing, China) for 40 min at room temperature. The targeted proteins were detected by using an enhanced chemiluminescence system (Millipore, Billerica, MA, United States). The quantification of band intensity was carried out using Image-Pro Plus software. Band densities were normalized to individual GAPDH internal control.

### RNA Extraction and Quantitative Real-Time PCR

Total RNA was isolated from LV tissues of rats (approximately 100 mg) using an Ultrapure RNA Kit (CWbio, Co., Ltd., Cat#CW0581) and reverse transcribed into cDNA using a PrimeScript™ RT Reagent Kit with gDNA Eraser (TaKaRa, Co., Ltd., Cat# RR047B) according to the manufacturer’s instructions. Real-time PCR reactions were carried out using the SYBR^®^ Premix Ex Taq™ II (Tli RNaseH Plus), ROX plus (TaKaRa, Co., Ltd., Cat# RR82LR) in an ABI 7500 thermal cycler (Thermo Fisher Scientific, Inc.). The amplification was performed as follows: 1 cycle of 95°C for 30 s and 45 cycles of 95°C for 5 s and 60°C for 40 s. Primers and probes were verified as operating at similar efficiencies. The levels of GAPDH expression were measured in all samples to normalize gene expression for sample-to-sample differences in RNA input, RNA quality, and reverse transcription efficiency. The fold differences in mRNA expression levels between samples were calculated using the 2-ΔΔ Ct relative quantification method. The primer sequences (Invitrogen Co., Ltd., Beijing, China) are as follows (forward and reverse, 5′-3′):

GAPDH, CCTTCCGTGTTCCTACCCC (forward) and GCCCAGGATGCCCTTTAGTG (reverse);
TNF-α, GGGCAGGTCTACTTTGGAGTCATTG (forward) and GGGCTCTGAGGAGTAGACGATAAAG (reverse);
TGF-β1, GAGAGCCCTGGATACCAACTACTGC (forward) and CAACCCAGGTCCTTCCTAAAGTCAA (reverse);
CTGF, GGGAAATGCTGTGAGGAGTGG (forward) and GCAGTTGGCTCGCATCATAGTT (reverse).

### Statistical Analysis

All data are expressed as mean ± standard deviation (SD). Statistical analysis was performed using one-way analysis of variance (ANOVA) followed by Tukey’s *post hoc* test or a repeated-measures ANOVA with the Bonferroni *post hoc* test for multiple comparisons. Changes of blood pressure in the same group were compared statistically by a paired *t* test. A probability of less than 0.05 was considered to be statistically significant.

## Results

### EA Alleviates Cardiac Fibrosis via Suppressing Elevated Blood Pressure in SHR

**FIGURE 1:**
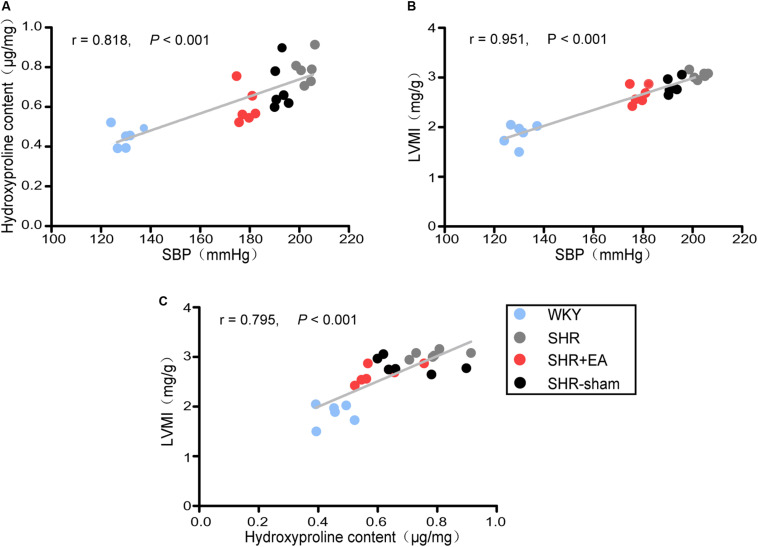
Correlation analysis in WKY rats and SHRs. Systolic blood pressure (SBP) was measured by the tail-cuff method after 8 weeks of EA treatment. The left ventricles were harvested for various analyses. The relationship of hydroxyproline (Hyp) content with SBP, left ventricular mass index (LVMI) with SBP, and LVMI with hydroxyproline content is shown in **(A)**, **(B)**, and **(C)**, respectively (*n* = 6 each group).

To access the cardiac fibrotic alteration following prolonged elevated blood pressure, the SBP, LVMI, and level of hydroxyproline in cardiac tissue were examined at 21 weeks, and their reciprocal correlations are presented in Figure 1. Our data shows that, compared to WKY rats, the LVMI and hydroxyproline level were significantly higher in SHRs and had positive correlations with the increased SBP (*P* < 0.001, Figures 1A,B), suggesting the development of cardiac fibrosis was likely to be attributed to enhanced blood pressure. The LVMI was correlated with the hydroxyproline content (*P* < 0.001, Figure 1C), suggesting that reduced cardiac interstitial fibrosis contributes to the decreased LVMI by 8 weeks EA at PC6 in SHRs. Notably, 8 weeks EA treatment remarkably lowered the elevated SBP as well as the increased LVMI and hydroxyproline level in SHRs while no obvious changes were found in the SHR-Sham group.

### Ameliorating Effects of EA on Myocardial Fibrosis in SHRs

#### EA Reduced the Accumulation of Collagen and the Level of Hydroxyproline in Myocardial Tissue of SHR

**FIGURE 2:**
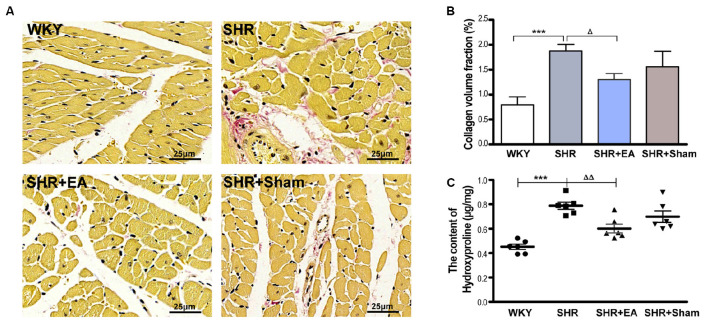
Effect of EA treatment on cardiac interstitial fibrosis in SHR. The tissue was taken after 8 weeks of EA treatment from SHR or at the same time course from WKY, and stained by Van Gieson staining. **(A)** Representative micrographs of cardiac collagen in the interstitial space of the left ventricle, which were ameliorated by EA treatment (bar = 25 μm). **(B)** Quantitative analysis of collagen volume fraction (CVF) by Van Gieson staining in myocardial tissues. ***P < 0.001 vs. WKY; ^Δ^*P* < 0.05 vs. SHR (*n* = 3 each group). **(C)** The hydroxyproline content (Hyp, μg/mg) was estimated after 8 weeks of EA treatment. ***P < 0.001 vs. WKY; ^ΔΔ^*P* < 0.01 vs. SHR (*n* = 6 each group).

The fibrotic alteration in myocardial tissue was assessed by Van Gieson staining. As shown in Figure 2A, there was excessive collagenic fiber hyperplasia in SHRs as characterized by enlarged, fractured, and disarranged myocardial fibers as well as hyperchromasia of light red cell components. As compared with SHR, these pathological damages were markedly restored in the SHR + EA group but showed less improvement in SHR + Sham group. Meanwhile, the CVF and level of Hyp in cardiac interstitial tissue were significantly increased in SHRs (Figures 2B,C, *P* < 0.001), which were attenuated by EA treatment (Figures 2B,C, *P* < 0.05, *P* < 0.01) rather than by sham EA. These results implied that EA at PC6 was effective in attenuating the myocardial fibrosis in SHRs.

### EA Lowered the Expression of Col I and Col III in Myocardial Tissue of SHR

**FIGURE 3:**
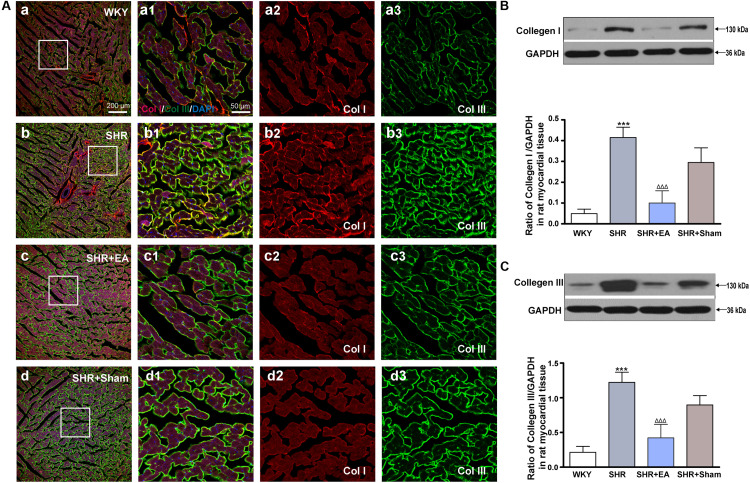
Effect of EA treatment on Collagen I and Collagen III deposition in the left ventricle myocardium. Spontaneously hypertensive rats (SHRs) were treated with EA for 8 weeks. **(A)** The SHR and WKY rats’ left ventricles were harvested for fluorescent immunohistochemical staining with Collagen I (red), Collagen III (green), and DAPI (blue). **(a–d)** Interstitial fibrosis in SHR myocardium documented by accumulation of fibrillary collagen I and collagen III. **(a1–d1)** The magnified photos from the areas of the boxes in **(a–d)**, respectively, showing the Collagen I and Collagen III in detail. **(a2–a3,b2–b3,c2–c3,d2–d3)** represent the tryptase **(a2–d2)** and phalloidin **(a3–d3)** distribution in **(a1–d1)**, respectively. Scale bar for **(a–d)** is shown in **(a)** (bar = 200 μm), for **(a1–a3,b1–b3,c1–c3)** shown in **(a1)** (bar = 50 μm). Western blotting analysis of Collagen I **(B)** and Collagen III **(C)** protein in left ventricular myocardium after 8 weeks of EA treatment. ***P < 0.001 vs. WKY; ^ΔΔΔ^ *P* < 0.001 vs. SHR (*n* = 3 each group).

Based on immunostaining results (Figure 3A) and quantitative analysis (Figures 3B,C), as compared with WKY, the expression of Col I and Col III in the cardiac interstitial tissue of SHR were significantly increased, suggesting the excessive deposition of the collagen and sign of myocardial fibrosis. However, as compared with SHR, the expression of Col I and Col III were reduced in the SHR + EA group (*P* < 0.001) rather than the SHR + Sham group. These results show that EA at PC6 attenuated myocardial fibrosis in SHR via downregulating the local expression of Col I and Col III.

### Involvement of Ang II, TGF-β_1_ in the EA-Induced Antihypertensive and Antifibrosis Effects

**FIGURE 4:**
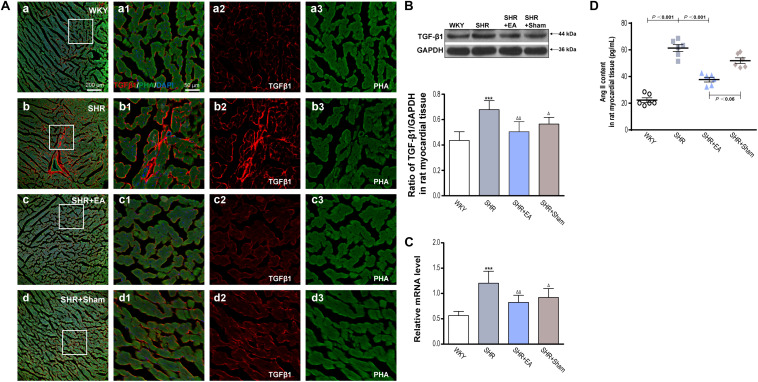
Effect of EA treatment on TGF-β_1_ deposition in the left ventricle myocardium. Spontaneously hypertensive rats (SHRs) were treated with EA for 8 weeks. **(A)** The SHR and WKY rats’ left ventricles were harvested for fluorescent immunohistochemical staining and histochemical staining with TGF-β_1_ (red), phalloidin (PHA, green), and DAPI (blue). **(a–d)** Fibrosis of the myocardium of SHRs and WKY rats as revealed by TGF-β_1_ (red) accumulation. **(a1–d1)** The magnified photos from the areas of the boxes in **(a–d)**, respectively, showing the TGF-β_1_ and myocardial structure in detail. **(a2-a3,b2-b3,c2-c3,d2-d3)** represent the TGF-β_1_ **(a2–d2)** and phalloidin **(a3–d3)** distribution in **(a1–d1)**, respectively. Scale bar for **(a–d)** is shown in **(a)** (bar = 200 μm), for **(a1-a3,b1-b3,c1-c3)** shown in **(a1)** (bar = 50 μm). Western blotting analysis of TGF-β_1_ **(B)** protein and real-time PCR analysis of TGF-β_1_ **(C)** mRNA in left ventricular myocardium after 8 weeks of EA treatment. ***P < 0.001 vs. WKY; ^Δ^ *P* < 0.05, ^Δ^ ^Δ^ *P* < 0.01 vs. SHR (*n* = 3 each group). **(D)** The concentration of TNF-αin myocardium measured by ELISA after 8 weeks of EA treatment (*n* = 6 each group).

Ang II, which has a great vasoconstrictive effect, plays an important role in myocardial interstitial fibrosis through TGF-β1. An enzyme-linked immunosorbent assay showed that the levels of Ang II in SHRs were significantly higher than WKY, in heart tissue (Figure 4D, *P* < 0.001). However, EA was effective in reducing the content of Ang II in SHRs (Figure 4D, *P* < 0.001), which might mediate the inhibitory effects of EA on myocardial hypertension and myocardial fibrosis. Immunofluorescent staining of myocardial tissue revealed that the fluorescence intensity of TGF-β_1_ in SHR was significantly higher than WKY (Figure 4A), which was consistent with the quantitative outcomes derived from Western blotting and real-time PCR tests, showing that the local expression of TGF-β_1_ was increased in SHRs (Figures 4B,C, *P* < 0.001). Notably, 8-week EA treatment mildly attenuated the level of TGF-β1 in the SHR + EA group (Figures 4B,C, *P* < 0.01). In addition, both fluorescence intensity and protein expression of TGF-β_1_ were also slightly reduced in the SHR+Sham group (Figures 4B,C, *P* < 0.05). These results show that EA attenuated hypertension and myocardial fibrosis via downregulating the expression of Ang II and TGF-β_1_.

### Effects of EA at PC6 on the Expression of CTGF in Myocardial Tissue of SHR

**FIGURE 5:**
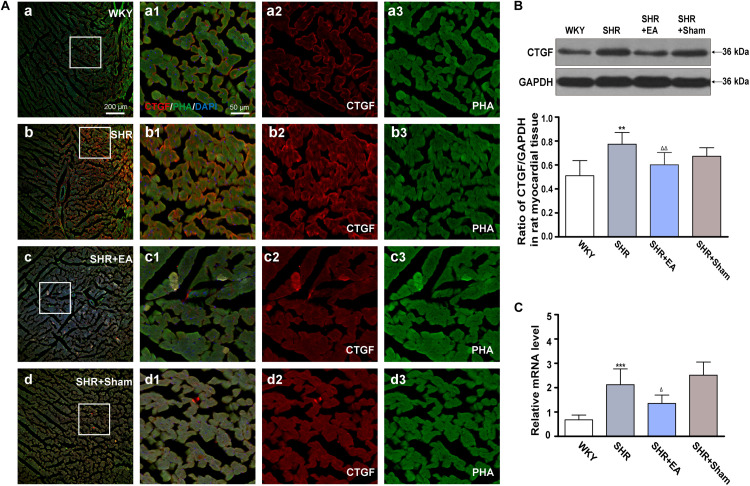
Effect of EA treatment on CTGF deposition in the left ventricle myocardium. Spontaneously hypertensive rats (SHRs) were treated with EA for 8 weeks. **(A)** The SHR and WKY rats’ left ventricles were harvested for fluorescent immunohistochemical staining and histochemical staining with CTGF (red), phalloidin (PHA, green), and DAPI (blue). **(a–d)** Fibrosis of the myocardium of SHR and WKY rats as revealed by TGF-β_1_ (red) accumulation. **(a1–d1)** The magnified photos from the areas of the boxes in **(a–d)**, respectively, showing the CTGF and myocardial structure in detail. **(a2-a3,b2-b3,c2-c3,d2-d3)** represent the CTGF **(a2–d2)** and phalloidin **(a3–d3)** distribution in **(a1–d1)**, respectively. Scale bar for **(a–d)** is shown in **(a)** (bar = 200 μm), for **(a1-a3,b1-b3,c1-c3)** shown in **(a1)** (bar = 50 μm). Western blotting analysis of CTGF **(B)** protein and real-time PCR analysis of CTGF **(C)** mRNA in left ventricular myocardium after 8 weeks of EA treatment. **P < 0.01, ***P < 0.001 vs. WKY; ^Δ^ *P* < 0.05, ^Δ^ ^Δ^ *P* < 0.01 vs. SHR (*n* = 3 each group).

Fluorescent immunohistochemical staining and histochemical staining revealed that the fluorescence intensity of CTGF in SHR was significantly higher than in WKY (Figure 5A). As compared with WKY, both Western blotting and real-time PCR revealed that local expression of CTGF was significantly increased in myocardial tissue of SHRs (Figures 5B,C, *P* < 0.01, *P* < 0.001), which were attenuated in the SHR + EA group (Figures 5B,C, *P* < 0.01, *P* < 0.05). These results showed that EA attenuated hypertension and myocardial fibrosis via downregulating the expression of CTGF.

### Involvement of TNF-α, MMP-2, and MMP-9 in the EA-Induced Antihypertensive and Antifibrosis Effects

**FIGURE 6:**
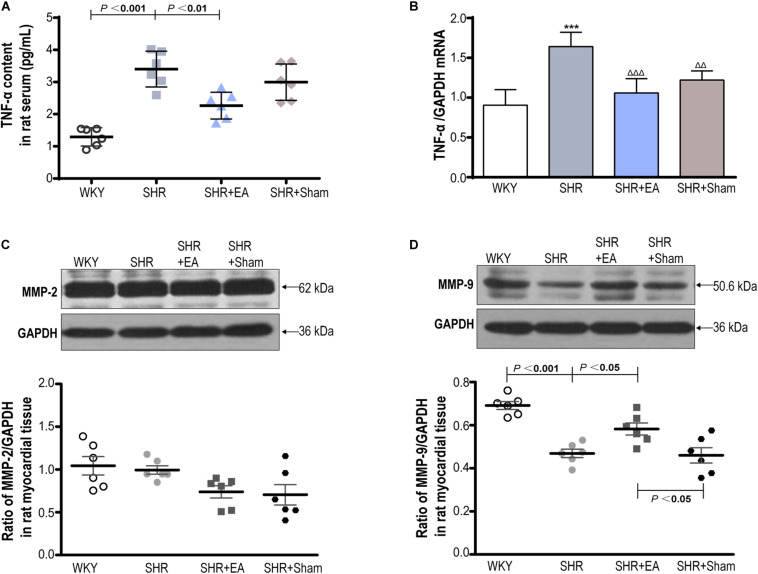
The roles of TNF-α, MMP-2, and MMP-9 in the cardiac protective effects of EA on SHR. The concentration of TNF-αin serum **(A)** measured by ELISA and heart tissue **(B)** measured by real-time PCR after 8 weeks of EA treatment. Western blotting analysis of MMP-2 **(C)** and MMP-9 **(D)** protein in left ventricular myocardium after 8 weeks of EA treatment. ***P < 0.001 vs. WKY; ^Δ^ ^Δ^ *P* < 0.01, ^ΔΔΔ^ *P* < 0.001 vs. SHR (*n* = 6 each group).

Enzyme-linked immunosorbent assay and real-time PCR showed that the local expression of TNF-α in SHR was significantly higher than in WKY in both serum and heart tissue (Figures 6A,B, *P* < 0.001). In addition, Western blotting revealed that local expression of MMP-9 in SHR was markedly lower than in WKY (Figure 6D, *P* < 0.001), whereas MMP-2 showed no difference among three groups (Figure 6C), and the expression of MMP-9 showed no difference between the SHR and SHR + Sham groups (Figure 6D). Notably, EA was effective in reversing the expression patterns of TNF-α and MMP-9 in SHR (*P* < 0.01, *P* < 0.001), which might mediate the inhibitory effects of EA on myocardial hypertension and myocardial fibrosis.

## Discussion

**FIGURE 7:**
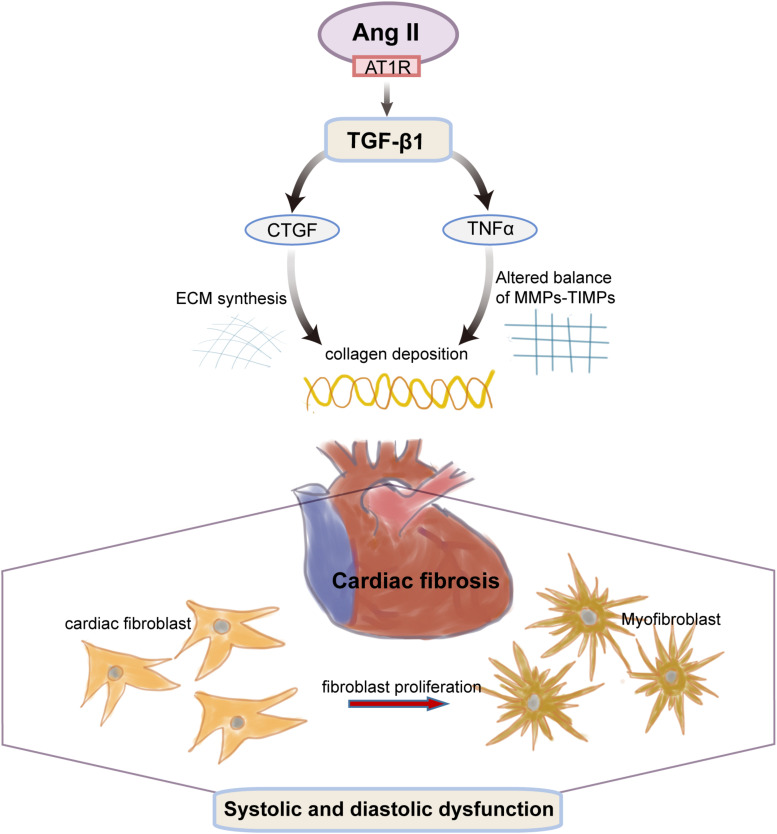
The transition of fibroblasts to myofibroblasts is an early event in hypertension-induced myocardial fibrosis. Cardiac fibroblasts can produce a number of active substances (such as Ang II, TGF-β_1_, CTGF, TNFα), which change the collagen deposition due to both a stiffening of the ECM synthesis and an altered balance of MMPs and their inhibitors (TIMP). The altered molecular and cellular events of the cardiac myocytes collectively result in progressive cardiac dysfunction.

Long-term hypertension is a common pathophysiologic condition in cardiac remodeling and plays a critical role in the pathogenesis of fibrotic cardiomyopathy in patients with primarily or genetically caused enhancement of blood pressure (Berk et al., 2007). Our previous results suggest that (Xin et al., 2017) the blood pressure of SHR from 13 to 21 weeks of age were sustained in the range of 170–200 mmHg and remained on an increasing trend with age while 8-week EA at PC6 was effective in lowering all phases of blood pressure in SHR rats. Therefore, MF induced by long-term hypertension is more likely to happen in SHRs of that age, which is suitable for the observation of the pathological process of hypertension-myocardial fibrosis in the present study (Figure 7).

Cardiac interstitial fibrosis, which is characterized by the collagen-based matrix network, contributes to both systolic and diastolic dysfunction in numerous cardiac pathophysiologic conditions (Kong et al., 2014). Collagen type I make up approximately 85% of total myocardial collagen while collagen type III accounts for about 11% of the total in the heart (Kania et al., 2009). It was also found that the abnormally increased content and out-of-proportion two different kinds of collagen could reflex the pathological alteration of MF in the hypertensive condition.

In the present study, we investigated the antifibrosis effects of EA at PC6 in SHRs and its underlying mechanisms. Our result is in alignment with previous reports (Wang et al., 2017; Chiang et al., 2018) showing that, as compared with WKY controls, the collagen fiber proliferated obviously, and the CVF and Hyp as well as the expression of Col I and Col III in cardiac interstitial tissue were significantly increased in SHRs, which indicated the pathological manifestations of MF. Of note is that the degeneration and structure disturbance of the myocardium were improved by 8 weeks of EA treatment.

It has been suggested that acupuncture at certain acupoints exerts antihypertensive effects, ameliorating heart damage induced by hypertension (Li et al., 2012a). PC6 is effective to improve cardiac function and is a benefit for treating various cardiovascular disorders including hypertension (Xin et al., 2017) and myocardial ischemia (Shi et al., 2017), which is attributed to its anatomical basis, segmental mechanisms (Li and Longhurst, 2010), and modulation on endocrine networks (Li et al., 2012b). In the last decades, several studies have focused on the neurophysiological bases of the effects of PC6 stimulation on cardiovascular mechanisms. Our previous study demonstrates that 8-week EA at PC6 was effective in lowering SBP in SHRs. In the present study, we showed that the Hyp content was positively correlated with the SBP. The LVMI was also positively correlated with the SBP. These results suggest that the reduction of blood pressure by EA at PC6 may contribute to its attenuation of cardiac fibrosis.

Ang II, through the ANG II type 1 receptor (AT1R) (Varo et al., 2000), causes vasoconstriction and elevated blood pressure and plays a causal role in myocardial interstitial fibrosis (Wu et al., 2002). More importantly, Ang II is also downstream of TGF-β_1_ to facilitate that pathological course (Shyu, 2017). TGF-β1 is a fibrogenic cytokine that may directly trigger the differentiation of cardiac fibroblasts to activated myofibroblasts to encode fibrillary collagen (Dobaczewski et al., 2011) and participate in the transition from stable hypertrophy to heart failure (Lim and Zhu, 2006). Meanwhile, it was also found that CTGF, as a downstream mediator of TGF-β_1,_ also has a great effect on fibroblasts induced by TGF-β_1_ (Varghese et al., 2017) and significantly upregulated in human heart failure and the myocardial fibrosis–associated animal model (Koshman et al., 2013). In addition, TNF-α, as a proinflammatory cytokine, promotes collagen deposition in fibrotic myocardium myocardia and ECM accumulation by affecting MMP expression and activity, which can lead to myocardial fibrosis (Leask, 2015). It has been shown that TNF-α suppressed the TGFβ_1_/Smad signaling pathway through its key effector molecule NF-kB, which activates the inhibitory Smad7 (Kassiri et al., 2009), making these two cytokines combined closely. It was also found that the co-cross-talk of them can enhance fibrotic change in TNF-α overexpressing animals and may involve fibroblast-mast cell interactions (Zhang et al., 2011). Furthermore, by searching the database, we can easily find that, in the pressure overload model of heart disease, MMP2-deficient mice showed reduced myocardial hypertrophy and fibrosis (Matsusaka et al., 2006) while MMP9 deficiency partially improved myocardial hypertrophy and fibrosis following pressure overload (Heymans et al., 2005). Overall, the Ang II-TGF-β_1_ pathway mediates the major pathophysiology of cardiac fibrosis, including cardiac fibroblast migration, proliferation, and collagen production (Chung et al., 2014).

Clinical studies suggest that antifibrotic activities of established drugs and compounds include Ang II inhibitors (De Mello and Specht, 2006) and TGF-β inhibitors as well as matrix metalloproteinase inhibitors (Spinale, 2007). Furthermore, aspirin, a non-selective epoxidase inhibitor, is becoming more and more popular in the clinic, and it can improve cardiac fibrosis by reducing the expression of TNF- α (Wu et al., 2012). However, the effective therapeutic strategies are still limited (Zile and Brutsaert, 2002). Therefore, developing new and effective approaches with minimum side effects to target cardiac disease processes linked to fibroblast function is an established therapeutic goal.

Previous studies from our lab and from other researchers uncovered the promising effects of acupuncture at PC6 on blood pressure control and heart protection (Huang et al., 2014; Gao et al., 2015; Xin et al., 2017). Remarkably, our previous work has endeavored the influential role of Angiotensin-converting enzyme (ACE), AT1R, and AngII type 2 receptor (AT2R) on the pathological progression from hypertension to cardiovascular remodeling in SHR by EA treatment. It has been demonstrated that acupuncture at PC6 is effective for protecting the myocardium from chronic ischemic injury by decreasing the serous Ang II concentration (Shi et al., 2017). EA was effective in regulating the levels of TNFα in animal models of ulcerative colitis and zymosan-induced acute arthritis (Park and Namgung, 2018). Additionally, acupuncture at “Quchi” (LI 11) and “Zusanli” (ST 36) probably intervenes in the process of renal interstitial fibrosis by reducing synthesis of kidney type I, type III collagen and restraining expression of TGF-β1 (Chen et al., 2013). However, there’s no evidence showing whether or not the Ang II-TGF-β1 pathway is involved in mediating the EA-induced antihypertensive and antifibrosis effects yet. The present study demonstrates that the levels of Ang II in SHRs were significantly higher than WKY in heart tissue. However, EA was effective in reducing the content of Ang II in SHRs. With fluorescent immunohistochemical staining and histochemical staining, Western blotting assay, and real-time PCR, we found that, in the SHR + EA group, the enhanced TGF-β_1_ and CTGF were attenuated after 8 weeks of EA treatment, suggesting their involvements in the mediation of the EA’s antihypertensive and antifibrosis effects on SHR.

On the other hand, enzyme-linked immunosorbent assay and real-time PCR, respectively, showed that, in the SHR + EA group, the elevated levels and local expression of TNF-α were significantly reduced in both serum and heart tissue. In addition, Western blotting revealed that EA was effective in reversing the expression patterns of MMP-9 in SHR, indicating their involvement in the mediation of the EA’s antihypertensive and antifibrosis effects on SHRs.

## Conclusion

Collectively, the results of present study suggest that 8 weeks of EA at PC6 inhibited myocardial fibrosis in SHRs, which might be mediated by downregulation of an enhanced Ang II -TGF-β1-CTGF/TNF-α pathway as well as upregulation of the diminished expression of MMP-9. Moreover, targeting the Ang II-TGF-β1 pathway by EA treatment could slow down fibrogenic heart disease process and may provide a promising therapeutic approach that could potentially lead to a less invasive and possibly more effective treatment in cardiovascular disorders caused by hypertension-induced myocardial fibrosis.

## Data Availability Statement

The raw data supporting the conclusions of this article will be made available by the authors, without undue reservation, to any qualified researcher.

## Ethics Statement

The animal study was reviewed and approved by the Committee on the Use and Care of Experimental Animals of Institute of Acupuncture and Moxibustion, China Academy of Chinese Medical Sciences.

## Author Contributions

The experiments were done by J-JX, J-HG, Q-FD, F-YL, Y-XZ, QL, J-JC, and D-SX. W-ZB and X-HJ provided advice on the statistical analyses and data interpretation. J-JX and X-CY drafted and finalized the manuscript. J-HG and X-CY were responsible for the conception, design, and supuervision of the implementation of the study. All authors contributed to the article and approved the submitted version.

## Conflict of Interest

The authors declare that the research was conducted in the absence of any commercial or financial relationships that could be construed as a potential conflict of interest.
